# Postpartum Management of Women Begun on Levothyroxine during Pregnancy

**DOI:** 10.3389/fendo.2015.00183

**Published:** 2015-11-30

**Authors:** Alex Stagnaro-Green

**Affiliations:** ^1^University of Illinois Rockford College of Medicine, Rockford, IL, USA

**Keywords:** pregnancy, thyroid, hypothyroidism, hyperthyroidism, postpartum thyroiditis, treatment

## Abstract

During pregnancy, the thyroid gland must produce 50% more thyroid hormone to maintain the euthyroid state. Women with decreased thyroid reserve preconception, most typically due to Hashimoto’s thyroiditis, may develop hypothyroidism during pregnancy. Data over the last 20 years have reported a strong association between subclinical hypothyroidism and adverse maternal/fetal events. As a result of this association, an increasing number of women are being screened for thyroid disease either preconception or at the first prenatal visit. Consequently, an ever increasing number of women are being initiated on levothyroxine for the first time during pregnancy. At present, there are very limited guidelines related to the management of the thyroid disease in these women postpartum. Based on an understanding of the physiology of the thyroid gland during pregnancy and postpartum, and the personal clinical experience of the author, recommendations for the postpartum management of women who were started on levothyroxine during pregnancy are presented.

Pregnancy has a profound impact on the thyroid gland and on thyroid function (1). Estrogen levels, which are elevated during pregnancy, result in a dramatic increase in thyroxine binding globulin. Increased thyroxine-binding globulin levels, transplacental transfer of thyroxine, and changes in deiodinase activity, necessitate a 50% rise in total thyroxine production so as to maintain adequate levels of free thyroid hormone (2). Women with a normal functioning thyroid gland remain euthyroid throughout pregnancy due to this compensatory mechanism. Pregnancy therefore teaches us that the healthy thyroid gland has significant reserve as it can accommodate up to the 50% increase in thyroid hormone production while maintaining the euthyroid state. On the other hand, women with decreased thyroidal reserve preconception, most typically due to Hashimoto’s thyroiditis, may develop subclinical hypothyroidism during pregnancy (3). Furthermore, women with unidentified subclinical hypothyroidism preconception may progress to overt hypothyroidism once pregnant. In essence, pregnancy serves as a stress test for the thyroid gland (Figure 1), bringing out hypothyroidism in women with underlying thyroid disease.

**Figure 1:**
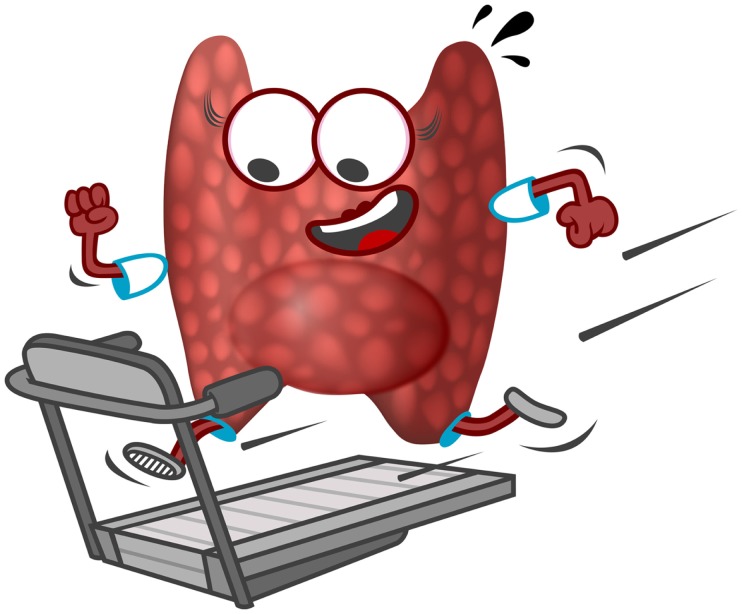
**Pregnancy-thyroid stress test**. Pictorial representation of the fact that during pregnancy, the thyroid gland mostly secretes 50% more hormone than the non-pregnant state, and therefore pregnancy represents a stress test for the thyroid.

Screening for thyroid disease during pregnancy, although still the subject of much debate (4), is increasing in frequency. The incidence of screening varies widely with surveys of members of thyroid associations around the world revealing that 21–69% of respondents support universal screening (5–7). The increase in screening mirrors accumulating evidence from observational studies that subclinical hypothyroidism is associated with multiple adverse maternal and fetal outcomes including miscarriage (8), gestational hypertension (9), gestational diabetes (10), and preterm delivery (11). Nevertheless, most professional societies do not recommend universal thyroid screening during pregnancy due to the limited and mixed results of studies prospectively evaluating the impact of levothyroxine treatment of subclinical hypothyroidism during pregnancy on maternal and fetal outcomes (12, 13). It is therefore not surprising that in 2011, the American Thyroid Association guidelines stated that, “There is insufficient evidence to recommend for or against universal TSH screening at the first trimester visit” (14), whereas the 2012 Endocrine Society Guidelines stated that, “The committee could not reach agreement with regard to screening recommendations for all newly pregnant women” (15). Similarly, the American College of Obstetricians and Gynecologists does not recommend universal screening (16). However, recently universal screening for thyroid disease during pregnancy is being recommended by some associations and researchers in the field based on the importance of detecting and treating overt hypothyroidism. The rationale is based on the frequency of overt hypothyroidism during pregnancy (incidence 0.5–1.0%) (17), the link to adverse outcomes, which can be prevented with levothyroxine therapy (18), and studies documenting its cost-effectiveness (19, 20). Proponents of universal thyroid screening during pregnancy include the Spanish Endocrine Society (21), the Indian Endocrine Society (22), and various researchers in the field (23, 24). Interestingly, the 2014 European Thyroid Association Guidelines do not recommend universal screening to detect subclinical hypothyroidism, but a majority of the authors recommended universal screening to detect overt hypothyroidism (25).

Thyroid dysfunction during pregnancy is common. As noted, earlier studies that screened all pregnant women for thyroid dysfunction identify 0.5–1.0% of women with overt hypothyroidism (elevated TSH and decreased free T4) (17). Studies screening for subclinical hypothyroidism (elevated TSH and normal free T4) have yielded an incidence that varies widely. Early studies, utilizing a TSH cutoff of >4.0 mIU/L, reported an incidence of SCH between 2 and 3% (11). However, recent studies, which utilized a TSH normal upper limit in the first trimester of 2.5 mIU/L, have reported an incidence as high as 15.5% (26).

Given the increased frequency with which women are screened for thyroid dysfunction during pregnancy and the incidence of OH and SCH, an ever increasing number of women are being identified for the first time with thyroid dysfunction during pregnancy. Furthermore, between 10 and 20% of all pregnant women are either thyroid peroxidase or thyroglobulin antibody positive (thyroid antibody positive) and euthyroid. Both the American Thyroid Association and Endocrine Society recommend that all thyroid antibody positive first trimester pregnant women with a TSH >2.5 mIU/L should be placed on levothyroxine. Both societies also recommend levothyroxine therapy in all pregnant women with a TSH >10.0 mIU/L, irrespective of the thyroid antibody status or free T4 level. The Endocrine Society also recommends levothyroxine therapy for first trimester antibody negative women with SCH whereas the American Thyroid Association concluded that there is insufficient evidence either for or against levothyroxine intervention in thyroid antibody negative women. Neither society recommends levothyroxine therapy in pregnant euthyroid women who are thyroid antibody positive. At present, only one prospective study has been published in this population, although the results of that study revealed a decrease in both miscarriage and preterm delivery (27) The American College of Obstetricians and Gynecologists recommends levothyroxine therapy for overt hypothyroidism during pregnancy but does not recommend levothyroxine treatment for SCH or thyroid antibody positivity in euthyroid women (16).

As there are disparate recommendations from medical societies regarding levothyroxine treatment for SCH during pregnancy, it would be instructive to know how often clinicians begin levothyroxine therapy once SCH is diagnosed. A number of surveys have been performed in which physicians are presented with case scenarios of thyroid dysfunction during pregnancy with the goal of identifying the conditions under which levothyroxine would be initiated (5–7, 28). In surveys of endocrinologists, the majority would initiate therapy in a women whose TSH exceeded 2.5 mIU/ml and who were thyroid peroxidase positive, whereas approximately half of the respondents would begin thyroid hormone if the TSH was above 2.5 mIU/ml in thyroid antibody negative women.

Given the above, it can be concluded that there are many women in which levothyroxine is initiated during pregnancy. Based on clinical guidelines, it can be assumed that endocrinologists are more likely to initiate therapy for SCH than obstetricians. As no professional society at this point recommends therapy for euthyroid thyroid antibody positive pregnant women, it can be concluded that the vast majority of women in this category do not receive levothyroxine. However, given the decreased miscarriage rates in euthyroid thyroid antibody positive women treated with levothyroxine reported by Negro et al. (28), it can be assumed that a percentage of women with multiple miscarriages who are euthyroid and thyroid antibody positivity are given levothyroxine. Finally, all women with OH identified during pregnancy receive levothyroxine.

An important clinical question regards the optimal management of hypothyroidism postpartum in women begun on levothyroxine therapy during pregnancy. This question has become increasingly relevant as more women have been identified with OH and SCH during pregnancy, and therefore, more women have been started on levothyroxine during gestation. This area has received limited attention by existing guidelines. Only the European Thyroid Association Guidelines consider this issue and do so with a focus solely on thyroid antibody negative pregnant women started on levothyroxine for a TSH <5 mIU/L (25). In this subset of women, the ETA Guidelines recommend to discontinue levothyroxine postpartum and check a TSH at 6 weeks postpartum. As no prospective studies have investigated this issue, the remainder of the article will define various postpartum scenarios and provide recommendations based on the physiology of the thyroid gland during pregnancy and postpartum and the clinical practice of the present author.

I utilize a number of principles to guide levothyroxine decisions postpartum. First of all, the higher the initial TSH during pregnancy at the time of diagnosis, the more likely that levothyroxine will be required to maintain euthyroidism postpartum. Second, and in many respects, a corollary of the first principle is the higher the dose of levothyroxine required during pregnancy to achieve euthyroidism, the more likely that levothyroxine will be required postpartum. Third, hypothyroid women who were found to be thyroid antibody positive during pregnancy are more likely to require levothyroxine postpartum than hypothyroid women who are thyroid antibody negative. Fourth, complicating postpartum management in thyroid antibody positive women is the possibility of postpartum thyroiditis (PPT). Finally, the article by Shields et al. is instructive as it presents thyroid function tests at 5 years postpartum in women who had subclinical hypothyroidism (TSH > 3 mIU/L) at 28 weeks gestation (29). The majority of women (75%) were euthyroid at 5 years, with persistent hypothyroidism (as determined by a TSH >4.5 mIU/L or ongoing levothyroxine therapy) seen more frequently in women who were thyroid antibody positive or had a TSH >5 mIU/L at 28 weeks gestation.

## Overt Hypothyroidism Diagnosed During Pregnancy – Irrespective of Thyroid Antibody Status

Women with overt hypothyroidism initially diagnosed during pregnancy comprise between 0.5 and 1.0% of pregnant women. The majority of these women will be thyroid antibody positive. Once diagnosed, the recommendation is to provide a dose of levothyroxine, which will optimize the TSH level in the shortest period of time. Abalovich et al. performed a retrospective study, which evaluated the dose of levothyroxine needed to treat different degrees of new onset hypothyroidism in pregnancy. Based on their data of 12 women, Abalovich and colleagues concluded that women with newly diagnosed overt hypothyroidism in pregnancy should be started on a dose of levothyroxine of 2.33 μg/kg/day (30). In general, my practice is to initiate therapy with a dose of ~100 μg/day and to reevaluate the regimen with a TSH at 4–6 weeks.

In deciding on a postpartum dose of levothyroxine, I postulate that at a minimum, the patient had subclinical hypothyroidism pregestation, which progressed to overt hypothyroidism during pregnancy. It is also feasible that the patient had overt hypothyroidism pregestation, which worsened once pregnancy was achieved. The final scenario, although least likely, is that the patient was euthyroid pregestation but had minimal ability, due to underlying Hashimoto’s disease, to compensate to the increased demand for thyroid production during pregnancy. Given all these scenarios, it is reasonable to assume that levothyroxine will be required postpartum to maintain euthyroidism. It can also be assumed that the postpartum dose of levothyroxine required following delivery is less than the dose needed to maintain euthyroidism in the third trimester. Consequently, I recommend that immediately postpartum, the levothyroxine dose be decreased to two-thirds of the final dose administered during gestation. Follow-up thyroid function tests should be performed at the first postpartum visit, which is typically at 6 weeks following delivery.

## Subclinical Hypothyroidism – Thyroid Antibody Positive

Women with subclinical hypothyroidism initially diagnosed during pregnancy comprise between 2 and 15% of pregnant women. Many, but not all, of these women will be thyroid antibody positive. Once diagnosed, the recommendation in thyroid antibody positive women of both the American Thyroid Association and Endocrine Society is to begin levothyroxine therapy. The study by Abalovich et al. based its initial treatment recommendations on the level of TSH at diagnosis and did not take thyroid antibody status into consideration. Specifically, the recommendation was to initiate levothyroxine at a dose of 1.20 μg/kg/day in women with a TSH ≤4.2 mIU/L and 1.42 μg/kg/day if the TSH is between 4.2 and 10.0 mIU/L (30). My practice is to begin treatment with a levothyroxine dose of between 50 and 75 μg/day and to reassess the TSH in 4–6 weeks.

In deciding on a postpartum dose of levothyroxine, I postulate that prior to pregnancy, the patient had underlying Hashimoto’s thyroiditis (based on the presence of thyroid antibodies during pregnancy) and was either euthyroid with limited reserve or already had subclinical hypothyroidism. At present, literature does not exist informing us what percentage of women diagnosed with subclinical hypothyroidism in gestation were euthyroid prior to pregnancy and what percentage already had subclinical hypothyroidism. In making a therapeutic decision for this group of women, I make treatment decisions under the assumption that it is best to avoid subclinical hypothyroidism in the immediate postpartum when the woman is lactating and adjusting to the rigors of life with a newborn. Therefore, I recommend that all women in this group be maintained on levothyroxine in the immediate postpartum. Once again, it can be assumed that the dose of levothyroxine required following delivery is less than the dose needed to maintain euthyroidism in the third trimester. Consequently, I would recommend that immediately postpartum, the levothyroxine dose be decreased to half of the final dose administered during gestation. Follow-up thyroid function tests should be performed at the first postpartum visit.

## Subclinical Hypothyroidism – Thyroid Antibody Negative

As noted in the prior section, women with subclinical hypothyroidism initially diagnosed during pregnancy comprise between 2 and 15% of pregnant women. Although the majority of these women will be thyroid antibody positive, a distinct minority will be thyroid antibody negative. These women may be truly thyroid antibody negative or may have become antibody negative as a result of the immunosuppressive impact of pregnancy. Management recommendations in this group of women differ, with the Endocrine Society recommending that all pregnant women with subclinical hypothyroidism receive levothyroxine irrespective of their thyroid antibody status, whereas the American Thyroid Association states that there is insufficient data to make a recommendation. As noted above, Abalovich et al. recommend the following levothyroxine algorithm to initiate therapy in pregnant women with new onset subclinical hypothyroidism – 1.20 μg/kg/day in women with a TSH ≤4.2 mIU/ml and 1.42 μg/kg/day if the TSH is between 4.2 and 10.0 mIU/ml. It is, therefore, likely that all of these women will be on a minimum of 50 mcg of levothyroxine daily at delivery. My practice is to begin with 25 μg/day of levothyroxine if the TSH is between 2.5 and 5.0 mIU/ml and 50 μg if the TSH exceeds 5.0 mIU/ml. Once the TSH approaches 10.0 mIU/ml, I recommend, at a minimum, 75 μg/day of levothyroxine.

In deciding on a postpartum course of therapy, I utilize as a guide the maximal dose of levothyroxine required during pregnancy to maintain the euthyroid state. In women who required a maximal dose of 25 mcg, I routinely discontinue levothyroxine immediately postpartum. When the maximal dose during pregnancy was 50 mcg of levothyroxine, I decrease the dose to 25 mcg immediately following delivery. In women who require a maximum pregnancy dose of 75 or 100 mcg, I initiate 50 mcg of levothyroxine immediately postpartum. In those rare circumstances where more than 100 mcg of levothyroxine is needed during gestation, I recommend the postpartum dose to be 50% of the maximum pregnancy dose. At 6-week follow-up, I evaluate TSH, free T4, and repeat thyroid antibodies, as some of these women probably were thyroid antibody positive pregestation.

## Isolated Hypothyroxinemia of Pregnancy

Isolated hypothyroxinemia (IH) of pregnancy is defined as a free thyroxine level below normal with a normal TSH level in a thyroid antibody negative woman. Whether or not to treat IH is even more controversial than whether or not to treat subclinical hypothyroidism. As there have been no studies demonstrating improved outcome in treating IH during pregnancy, I do not begin levothyroxine in this group of women. This is consistent with the American Thyroid Association Guidelines, which do not recommend levothyroxine for IH of pregnancy (14). However, both the Endocrine Society Guidelines and the European Thyroid Association Guidelines state that levothyroxine intervention can be considered, especially for IH diagnosed in the first trimester (15, 25). In those instances where levothyroxine is initiated during pregnancy, my recommendation would be to discontinue the levothyroxine immediately postpartum. Follow-up testing postpartum is not indicated.

## Impact of Postpartum Thyroiditis

Postpartum thyroiditis is defined as thyroid hormone dysfunction in the initial postpartum year in women who were euthyroid prior to pregnancy (31). In its classic triphasic form, a hyperthyroid phase occurs between 1 and 3 months postpartum, followed by a hypothyroid phase between 3 and 9 months postpartum, with a return to the euthyroid state by the end of the first postpartum year. The vast majority of women who develop PPT are thyroid antibody positive prior to pregnancy. Interestingly, two studies have demonstrated that PPT can also develop in women who had Hashimoto’s thyroiditis prior to pregnancy and who already were being treated with levothyroxine (32, 33). For PPT to occur in this group of women, it must be assumed that although levothyroxine is needed to retain the euthyroid state, the woman still had residual functioning thyroid tissue. It is therefore the remaining thyroid tissue that experiences thyroiditis in the postpartum resulting in alterations in thyroid function.

The potential for PPT complicates the postpartum management of thyroid antibody positive women who are first identified during pregnancy as requiring thyroid hormone replacement. As this represents the majority of women who will begin levothyroxine during gestation, the postpartum management must take into account the possibility of PPT. This could result in a scenario in which a woman started on levothyroxine during gestation for subclinical hypothyroidism presents with hyperthyroidism in the first couple of months, only to be followed by hypothyroidism (potentially quite pronounced) later in the first postpartum year. Furthermore, many women with PPT present solely with either a hyperthyroid or hypothyroid phase Therefore, it is critical to evaluate for PPT when managing postpartum women who were started on levothyroxine during pregnancy.

## Conclusion

In conclusion, due to an increased appreciation of the relationship between thyroid disease and maternal/fetal adverse outcomes, the number of women identified during pregnancy with thyroid dysfunction and initiated on levothyroxine therapy is increasing. The postpartum management of these women depends on multiple factors including the degree of hypothyroidism at diagnosis, the maximum dose of levothyroxine required to maintain the euthyroid state during pregnancy, the presence or absence of thyroid antibodies, and whether or not PPT occurs. The recommendations included within this article (Table 1) are based on the physiological changes, which occur during pregnancy and the postpartum to the thyroid gland, and the clinical experience of the author. Prospective management trials are needed to evaluate the recommendations made within this manuscript and to determine optimal treatment of thyroid disease postpartum in women who had levothyroxine initiated during pregnancy.

**Table 1:** **Recommendations for the postpartum management of levothyroxine in women who had levothyroxine initiated during pregnancy**.

Diagnosis in pregnancy	Postpartum treatment recommendation	Postpartum testing
Overt hypothyroidism	2/3 of final LT4 dose	6 weeks
Subclinical hypothyroidism	1/2 of final LT4 dose	6 weeks
Thyroid antibody positive		
Subclinical hypothyroidism	If last dose 25 mcg, D/C LT4 postpartum	6 weeks
Thyroid antibody negative	
	If last dose 50 mcg, start 25 mcg postpartum	6 weeks
	If last dose 75–100 mcg, start 50 mcg postpartum	6 weeks
	If last dose >100 mcg, start 50 mcg/day final dose	
Isolated hypothyroxinemia	D/C LT4 postpartum	Not recommended

## Conflict of Interest Statement

The author declares that the research was conducted in the absence of any commercial or financial relationships that could be construed as a potential conflict of interest.
